# Stereotactic body radiation therapy for the treatment of lymph node metastases: a retrospective mono-institutional study in a large cohort of patients

**DOI:** 10.3389/fonc.2023.1163213

**Published:** 2023-08-03

**Authors:** Donatella Caivano, Paolo Bonome, Donato Pezzulla, Margherita Rotondi, Riccardo Carlo Sigillo, Vitaliana De Sanctis, Maurizio Valeriani, Mattia Falchetto Osti

**Affiliations:** ^1^ Department of Medical and Surgical Sciences and Translational Medicine - Sant’ Andrea Hospital, Sapienza University of Rome, Rome, Italy; ^2^ Radiotherapy, Santa Maria Goretti Hospita, Latina, Italy; ^3^ Radiation Oncology Unit, Gemelli Molise Hospital - Università Cattolica del Sacro Cuore, Campobasso, Italy; ^4^ Department of Radiation Oncology, Sant’ Andrea Hospital, Sapienza University of Rome, Rome, Italy

**Keywords:** SBRT, lymph nodes metastasis, oligometastatic disease, ablative radiotherapy, metastasis direct therapy

## Abstract

**Introduction:**

Lymph node metastases (NMs) are a common site of tumor spread that can occur at different times of the disease. Stereotactic body radiation therapy (SBRT) can be a therapeutic option for the treatment of NMs in the setting of oligometastatic disease (OMD). The aim of this study was to evaluate as primary end points the local control (LC) and secondary end points the locoregional nodal control (LRNC), distant nodal control (DNC), distant metastasis-free survival (DMFS), progression-free survival (PFS), and overall survival (OS), and concurrently to assess the predictive factors of response.

**Methods:**

This is a retrospective study that analyzes a group of patients treated with SBRT on NMs from different primary tumors, with a of maximum five metastasis. Treated lesions were divided into four groups: oligometastatics, oligorecurrents, oligoprogressives, and oligopersistents.

**Results:**

From 2007 to 2021, 229 NMs were treated in 174 patients with different primary tumor. The schedule most represented was 30 Gy in five fractions. The LC was obtained in 90% of NMs treated by SBRT with rates at 1, 3, and 5 years of 93%, 86%, and 86%, respectively. The LRNC was reached in 84% of cases with rates at 1, 3, and 5 years of 88%, 83%, and 77%, respectively. The DNC was obtained in 87% of cases with rates at 1, 3, and 5 years of 92%, 82%, and 78%, respectively. The DMFS was obtained in 38% of cases with rates at 1, 3, and 5 years of 57%, 40%, and 30%, respectively. The rate of PFS were 44%, 23%, and 13% at 1, 3, and 5 years, respectively. The rates at 1, 3, and 5 years of OS were 78%, 48%, and 36%, respectively.

**Conclusion:**

SBRT is an option for the treatment of NMS, with high rates of LC, improving survival, and with a good safety and tolerance. Tumor volume, tumor burden, lesion site, and doses can be predictive factors of response; however, multi-institutional studies with a greater number of patients could be helpful to better select patients and understand the right integrations between ablative treatment and systemic therapies.

## Introduction

The oligometastatic disease (OMD) is an area of great interest, rapidly evolving in the characterization and treatment, with a large spectrum of presentation and behavior (1, 2). The first definition of OMD was introduced by Hellman and Weichselbaum in 1995 as an intermediate stage between limited disease and metastatic dissemination (3). In recent years, there has been a big effort to standardize the nomenclature; the proposal was the execution of reproducible trials and a better understanding of the disease. The European Society for Radiotherapy and Oncology (ESTRO) and the American Society for Radiation Oncology (ASTRO) have created a collaborative project to develop a consensus on patient identification and treatment (4–6).

Isolated lymph node metastases (NMs) represent sometimes the only site of disease, as an OMD. The lymph nodes are a common site of tumor spread that can occur at different times of the disease. The incidence of NMs is heterogeneous, and it depends on several factors, based on the primary tumor site, stage, histology, and grading. In the past, they were considered a marker of extensive disease, and systemic therapy was the standard of care; however, over recent years, a more radical approach is emerging in the oligometastatic setting for nodal lesions, with stereotactic body radiotherapy (SBRT) as one of the main weapons (7–10).

During recent decades, SBRT has become a new local treatment option for OMD as a metastasis direct therapy. This technique uses specialized technology able to give high doses of radiation in few fractions on the target, sparing normal tissue compared to conventional radiotherapy. In potentially operable cases, SBRT is more precise and conformed with less morbidity, invasiveness, and costs (11–13); however, lymph node metastases have some characteristics as the minimum organ motion, small volumes, well-defined contours, and a regular form for which they are an ideal target for SBRT treatments (14). This technique is very promising for OMD or in some primary tumors, with high rates of local control (LC) (15, 16). A Phase II trial, SABR-COMET, has indicated in OMD good results in terms of overall survival (OS) and progression-free survival (PFS). Long-term outcomes have shown an 8-year OS of 27.2% in the SBRT plus standard of care arm (SOC) vs. 13.6% in the SOC arm (17, 18).

The aim of this study was to evaluate in our cohort of patients treated at Sant’Andrea Hospital by SBRT on NMs as primary end points the rates of LC and secondary end points the rates of locoregional nodal control (LRNC), distant nodal control (DNC), distant metastasis-free survival (DMFS), progression-free survival (PFS), and OS, and concurrently to assess the predictive factors of response.

## Materials and methods

This is a retrospective study that analyzes a group of patients treated by SBRT on NMs from different primary tumors. The cases were discussed and approved by an internal tumor board. The criteria of inclusion are the following: ECOG performance status ≤2; primary cancer disease under control; maximum of five metastases of which one is the lymph node treated by SBRT; and maximum lymph node diameter ≤5 cm. A written informed consent was obtained from all patients before SBRT treatments. In addition, concomitant treatments were allowed. OMD is defined as a disease with a limited number of metastases detected on imaging (from 1 to 5) and is classified according to the patient’s history of metastasis and systemic therapies. The classifications were as follows: oligometastatics in case of lesions present at the moment of diagnosis; oligorecurrents in case of lesions observed during a free interval from systemic therapy; oligoprogressives in case of isolated progression during systemic therapy; and oligopersistents in case of isolated persistence of disease after systemic therapy.

### Treatment plan

All patients underwent a computed tomography (CT) simulation and were immobilized in the supine position. It was important that the position was stable, reproducible, and comfortable during the CT simulation and then during the irradiation. A 4DCT system was used to generate an internal target volume (ITV) to evaluate respiratory excursion when the localization of NMS needed the evaluation of excursion breathing. The ITV was created on the maximum intensity projection (MIP). The gross tumor volume (GTV) was the visible lesion on CT, but when the lesion was not well visible, we did an image fusion with diagnostic images, as an MR or CT/PET, in all scan sets. The sum of GTV plus ITV was expanded by 4 mm in all directions to create the planning tumor volume (PTV). In the other site, the clinical target volume (CTV) was equal to GTV, and PTV was the CTV plus 4 mm in all directions.

The contour and plan were performed with Eclipse v16 treatment planning system, and the algorithm used was AAA. The SBRT plan has had an implementation over the years. In 2008, a plan was created with a 3D conformational technique; then from 2007 to 2018, 159 plans were created with IMRT, and from 2018 to 2021, 69 plans were done with Rapid-Arc. Cone-beam CT was obtained prior to each treatment to check correct pre-treatment positioning.

The choice of single vs. multiple fractions was based on the volume of lesions and on the proximity of the target to critical organs. The doses were measured also in terms of biological effective dose (BED) assuming that the α/β ratio is 10 for tumor. The characteristics of lesions, doses, and treatments are summarized in **Tables 1** and **2**.

**Table 1:** The characteristics of lesions, doses, and treatments.

	Range	Number	Percentage	Medium	Median
Diameter of CTV (cm)	0.2–4.9			2.2	2.1
Volume of CTV (cc)	0.08–0.3			8.3	4.8
BED 10 (Gy)	33.6–120			74.7	76
EQD2 (Gy)	28–126			63	64
Total dose (Gy)	14–76			39.5	36
Fractionations	1–8				
Prescription dose (%)	91–100			97	97
Radiation therapy method					
Rapid arc	69				
IMRT	159				
3DCRT	1				
Localization of NMs treated					
Mediastinum		99	44%		
Pelvis		73	31%		
Abdomen		43	19%		
Other*		14	6%		
Time interval from diagnosis of tumor to SBRT (months)	2–393			60	42
Metastatic Status**					
Oligometastatic		8	3%		
Oligorecurrent		111	49%		
Oligoprogressive		93	40%		
Oligoperistent		16	7%		
Not defined		1	1%		
Number of metastasis at moment of SBRT					
1		134	58%		
2		73	33%		
3		16	7%		
4		3	1%		
5		3	1%		
Extra regional metastasis at moment of SBRT					
0		196	85%		
1		24	10%		
2		7	3%		
3		1	1%		
4		1	1%		
Sites of extra regional metastasis at moment of SBRT					
Visceral		23	10%		
Bone		9	4%		
Boths		2	1%		
Extra regional nodal disease at moment of SBRT					
0		200	87%		
1		26	11%		
2		3	2%		
Number of systemic therapies before SBRT					
0		61	26%		
1		106	46%		
2		45	20%		
3		11	5%		
4		4	2%		
Not defined		2	1%		
Concomitant systemic therapy					
Yes		77	34%		
No		152	66%		
Systemic therapy during SBRT					
Chemotherapy		14	6%		
Target therapy		14	6%		
Immunotherapy		4	2%		
Hormone therapy		41	18%		
Chemotherapy+target therapy		2	1%		
Target therapy+ hormone therapy		2	1%		
Number of systemic therapies after SBRT					
0		87	38%		
1		130	56%		
2		4	2%		
3		2	1%		
Not defined		6	3%		
Diagnosis with biopsy of lymph node treated					
Yes		3	2%		
No		225	97%		
Not specified		1	1%		
Technique of imaging used for diagnosis of lymph node treated					
CT scan		4	3%		
PET CT		225	97%		

*Others: axillary, supraclavicular nodes, subclavearies, and internal breast chain lymph nodes.

**Oligometastatic: metastasis at moment of diagnosis (≤5 lesions). Oligorecurrent: new lesion after free time from treatment. Oligoprogressive: new lesion during systemic treatment. Oligopersisten: persistent metastasis after systemic therapy.

**Table 2:** Distributions of doses, primary tumor, and tumor burden divided by site of treated lesions.

Lesion site		Pelvis	Abdomen	Thorax
N		72	44	99
Total dose (Gy)/no. of fractions (fx)	8/1fx	1	0	0
	10/1fx	1	1	0
	12/1fx	2	1	0
	14/1fx	3	0	0
	15/1fx	1	0	0
	16/1fx	3	1	0
	23/1fx	7	5	18
	24/6fx	5	0	0
	25/5fx	7	0	2
	27/3fx	0	0	1
	30/5fx	20	13	15
	32/4fx	1	0	0
	32.5/5fx	1	0	1
	35/5fx	3	0	3
	36/6fx	4	5	4
	37.5/5fx	1	1	1
	40/5fx	1	0	8
	44/8fx	0	1	0
	45/3fx	3	4	0
	48/8fx	6	6	17
	50/5fx	0	0	1
	52/8fx	0	0	4
	54/3fx	1	0	0
	56/8fx	1	0	3
	60/8fx	1	4	14
Primary tumor histology	Prostate	44	12	5
	Lung	1	0	50
	Lung+Other Tumor	0	0	6
	Breast	0	0	11
	Colon	6	7	4
	Cervix	5	2	0
	Other	16	23	23
Tumor burden	≤1 lesion	43	24	51
	>1 lesion	29	20	48

### Follow-up

The first clinical follow-up was at 2 months, then every 3 months for 2 years, and then every 6 months. Clinical evaluation and diagnostic imaging (CT or PET scan) were planned at physician’s choice. The criteria for radiological response evaluation were extrapolated from the RECIST 1.1 scale (Solid Tumors Response Evaluation Criteria). Toxicity was classified by the CTCAE scale v.4.4.

### Statistical analysis

The first end point of our study was to assess the LC and predictive factors of response; the secondary end points were to evaluate the LRNC, DNC, DMFS, PFS, and overall survival, and concurrently the predictive factors of response. The LC was defined as the time from the beginning of SBRT to the date of in-field progression or last follow-up. The LRNC is defined as the time from SBRT to the appearance of new locoregional lymph node metastases or last follow-up. The DNC is defined as the time from SBRT to the appearance of new non-regional lymph node metastases. The DMFS is defined as the time from the treatment to the appearance of non-nodal metastases or last follow-up. The PFS is defined as the time from the treatment to the progression of disease or last follow-up. The OS is calculated from the SBRT to either death or last follow-up.

To analyze actuarial outcomes, the Kaplan–Meier method was used; differences among subgroups were evaluated by log-rank tests. Cox logistic regression was used to carry out the univariate and multivariate analysis of factors predicting LC and LRNC on “a per lesion” basis and DMFS, DNR, PFS, and OS on “a per patient” basis. The result of the logistic regression model was expressed as odds ratios with 95% confidence intervals. Statistically significance was obtained if p<0.05. Only the variables with a p < 0.2 at univariate analysis were selected for the multivariate analysis in order to select the most relevant variables for this analysis.

Statistical analysis was carried out by SPSS statistical software (IBM Corp., released 2011, IBM SPSS Statistics for Windows, Version 20.0, Armonk, NY: IBM Corp).

## Results

### Baseline characteristics

From November 2007 to September 2021, 229 NMs were treated in 174 patients. The median age was 68 years (range, 24–90 years). We had 64% male and 36% female patients. The primary tumor most represented was lung in 54 patients and prostate in 39 patients. We had various primary tumors and different histologies; the characteristics of the patients are reported in **Table 3**. We had 8 oligometastatic, 111 oligorecurrent, 93 oligoprogressive, and 16 oligopersistent lesions; one was not defined. In 34% of the treatment, concomitant treatment was allowed. The medium volume of lesions was 8.3 cc (range, 0.08–60.3 cc), the medium diameter was 2.2 cm (range, 0.2–4.9 cm) (**Table 1**). The single and multiple fractions were administered in 22% and 78% of cases, respectively. The schedule most represented was 30 Gy in five fractions, and the medium BED (10) of SBRT was 74.7 Gy (range, 33.6–120 Gy).

**Table 3:** The characteristics of the patients.

	Range	Number	Percentage	Medium	Median
Gender					
Male		112	64%		
Female		62	36%		
Median age (years)	24–90			67	68
Performance status	0–2				
0		125	74%		
1		47	24%		
2		3	2%		
Primary tumor					
Lung		54	31%		
Prostate		39	22%		
Colon–rectum		18	10%		
Gynecological cancer		16	9%		
Breast		11	6%		
Upper GI		13	7%		
GU		9	5%		
HN		4	2%		
Others*		10	5%		
Histology					
Adenocarcinoma		107	61%		
SCC		11	6%		
SCLC		4	2%		
IDC		6	3%		
Others*		46	26%		

*Others histology: melanoma, clear cell tumor, ovarian carcinoma, neuroendocrine carcinoma, GIST, hepatocarcinoma, poorly differentiated carcinoma, papillary thyroid carcinoma, urethral carcinoma, sarcoma, without biopsy, not specified.

### Clinical outcomes

**Figure 1:**
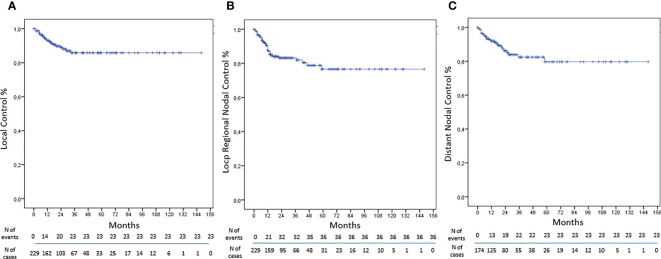
Kaplan–Meier curves: **(A)** LC, **(B)** LRNC, and **(C)** DNC.

The LC was obtained in 90% of NMs treated by SBRT with rates at 1, 3, and 5 years of 93%, 86%, and 86%, respectively (**Figure 1**). At univariate analysis, only the total dose >30 Gy was a statistically significant negative prognostic factor for LC (HR, 3.361; 95%CI, 1.323–8.534; p-value, 0.011); however, these data were not confirmed at multivariate analysis (**Table 4**).

**Table 4:** Univariate and multivariate Cox regression analysis of variables predicting LC on “lesion” basis.

		Univariate			Multivariate		
Variable	N.	Hazard ratio	95%CI	p-value	Hazard ratio	95%CI	p-value
**Age, years** <68 >68	121 108	1 1.750	0.758–4.044	**0.190**	1 0.460	0.173–1.229	0.121
**Sex** Male Female	150 79	1 1.615	0.708–3.684	0.255			
**Burden** 1 lesion More than 1 lesion	112 62	1 1.139	0.446–2.907	0.786			
**Lesion site** Thorax Pelvis Abdomen Other	99 72 43 15	1 0.484 0.935 0.496	0.170–1.375 0.329–2.667 0.065–3.817	**0.173** 0.900 0.501	1 0.918 0.625 1.368	0.106–7.963 0.065–6.024 0.149–12.597	0.938 0.684 0.782
**Total dose** ≤30 Gy >30 Gy	115 114	1 3.361	1.323–8.534	**0.011**	1	0.108–1.301	0.0122
**BED10** ≤75.9 Gy >75.9 Gy	126 103	1 1.760	0.771–4.015	**0.179**	1 1.357	0.414–4.442	0.614
**CTV** ≤4.82cc >4.82cc	114 115	1 1.619	0.708–3.701	0.253			
**Dose prescription** ≤97% >97%	143 86	1 0.515	0.203–1.308	**0.163**	1 2.110	0.720–6.180	0.173

The LRNC was reached in 84% of cases with rates at 1, 3, and 5 years of 88%, 83%, and 77% respectively (**Figure 1**). At univariate analysis abdominal localization was a positive statistically significant prognostic factor for LRNC (HR, 0.170; 95%CI, 0.040–0.720; p-value, 0.016), but not confirmed at multivariate analysis (**Table 5**).

**Table 5:** Univariate and multivariate Cox regression analysis of variables predicting LRNC on “lesion” basis.

		Univariate			Multivariate		
Variable	N.	Hazard ratio	95%CI	p-value	Hazard ratio	95%CI	p-value
**Age, years** ≤68 >68	121 108	1 0.914	0.473–1.766	0.789			
**Sex** Male Female	150 79	1 0.630	0.296–1.339	0.230			
**Burden** 1 lesion More than 1 lesion	112 62	1 1.377	0.614–3.086	0.437			
**Lesion site** Thorax Pelvis Abdomen Other	99 72 43 15	1 0.481 0.170 0	0.230–1.007 0.040–0.720 0.001–999999	**0.052** **0.016** 0.977	1 17434 7194 27173	0.001–999999 0.001–999999 0.001–999999	0.899 0.908 0.920
**Total dose** ≤30 Gy >30 Gy	115 114	1 1.449	0.750–2.802	0.270			
**BED10** ≤75.9 Gy >75.9 Gy	126 103	1 0.925	0.476–1.794	0.817			
**CTV** ≤4.82cc >4.82cc	114 115	1 0.731	0.373–1.430	0.360			
**Dose prescription** ≤97% >97%	143 86	1 0.571	0.275–1.185	**0.133**	1 1.060	0.465–2.414	0.890

The DNC was obtained in 87% of cases with rates at 1, 3, and 5 years of 92%, 82%, and 78%, respectively (**Figure 1**). At univariate analysis, the abdominal lesion site was a positive statistically significant prognostic factor for DNC (HR, 3.143; 95%CI, 1.138–8.679; p-value, 0.027), but not confirmed at the multivariate one. Furthermore, at univariate analysis, the volume of CTV > 4.82 cc was a statistically significant positive factor of DNC (HR, 0.310; 95%CI, 0.122–0.789; p-value, 0.014), and the multivariate analysis has confirmed these data (HR, 0.299; 95%CI, 0.111–0.805; p-value, 0.017) (**Table 6**).

**Table 6:** Univariate and multivariate Cox regression analysis of variables predicting DNC on “patient” basis.

		Univariate			Multivariate		
Variable	N.	Hazard ratio	95%CI	p-value	Hazard ratio	95%CI	p-value
**Age, years** ≤68 >68	89 86	1 1.311	0.575–2.991	0.52			
Sex							
Male Female	112 63	1 2.188	0.963–4.968	**0.061**	1 1.358	0.542–3.401	0.513
**Burden** 1 lesion More than 1 lesion	112 62	1 0.574	0.212–1.553	0.274			
Lesion site							
Thorax Pelvis Abdomen Other	81 51 31 12	1 0.912 3.143 2.977	0.289–2.882 1.138–8.679 0.769–11.531	0.876 **0.027** 0.114	1 0.915 2.544 4.124	0.278–3.009 0.903–7.167 1.020–16.670	0.884 0.077 **0.047**
**Total dose** ≤30 Gy >30 Gy	81 94	1 1.128	0.496–2.565	0.774			
**BED10** ≤75.9 Gy >75.9 Gy	85 90	1 0.814	0.357–1.859	0.626			
CTV							
≤4.82cc >4.82cc	76 99	1 0.310	0.122–0.789	**0.014**	1 0.299	0.111–0.805	**0.017**
**Dose prescription** ≤97% >97%	104 71	1 0.833	0.360–1.926	0.67			

**Figure 2:**
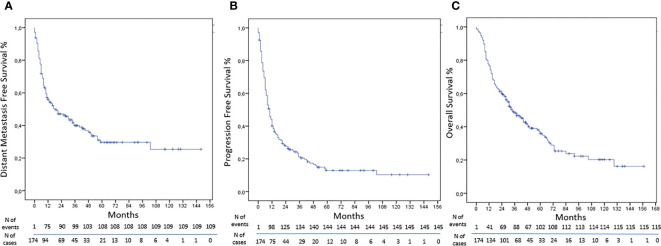
Kaplan–Meier curves: **(A)** DMFS, **(B)** PFS, and **(C)** OS.

The DMFS was obtained in 38% of cases with rates at 1, 3, and 5 years of 57%, 40%, and 30%, respectively (**Figure 2**). At univariate analysis, a higher tumor burden was a statistically significant predictor of worse results (HR, 1.738; 95%CI, 1.184–2.552; p-value, 0.005), and the same was confirmed at multivariate analysis (HR, 1.897; 95%CI, 1.273–2.828; p-value, 0.002). At univariate analysis, the CTV>4.82cc (HR, 1.689; 95%CI, 1.146–2.489; p-value, 0.008) and the total dose> 30 Gy (HR, 1.559; 95%CI, 1.064–2.285; p-value, 0.023) were statistically significant unfavorable prognostic factors for DMFS, but at multivariate analysis, only the BED>75.9Gy was a statistically significant negative prognostic factor (HR, 1.681; 95%CI, 1.132–2.497; p-value, 0.010) (**Table 7**).

**Table 7:** Univariate and multivariate Cox regression analysis of variables predicting DMFS on “patient” basis.

		Univariate			Multivariate		
Variable	N.	Hazard ratio	95%CI	p-value	Hazard ratio	95%CI	p-value
Age, years							
<68 >68	89 86	1 0.696	0.475–1.019	**0.062**	1 0.715	0.485–1.053	0.09
**Sex** Male Female	112 63	1 1.014	0.685–1.502	0.945			
Burden							
1 lesion More than 1 lesion	112 62	1 1.738	1.184–2.552	**0.005**	1 1.897	1.273–2.828	**0.002**
**Lesion site** Thorax Pelvis Abdomen Other	81 51 31 12	1 0.752 0.697 0.728	0.486–1.164 0.399–1.217 0.313–1.694	0.201 0.204 0.461			
Total dose							
≤30 Gy >30 Gy	81 94	1 1.559	1.064–2.285	**0.023**	1 1.463	0.909–2.354	0.117
BED10							
≤75.9 Gy >75.9 Gy	85 90	1 1.375	0.942–2.006	**0.099**	1 1.681	1.132–2.497	**0.01**
CTV							
≤4.82cc >4.82cc	76 99	1 1.689	1.146–2.489	**0.008**	1 1.236	0.765–1.999	0.387
**Dose prescription** ≤97% >97%	104 71	1 1.144	0.779–1.679	0.493			

The rate of PFS were 44%, 23%, and 13% at 1, 3, and 5 years, respectively (**Figure 2**). At univariate analysis, a pelvic lesion site was a favorable prognostic factor (HR, 0.565; 95%CI, 0.381–0.839; p-value, 0.005), but the data were not confirmed at multivariate analysis. On the other hand, a CTV>4.82cc was a negative statistically significant prognostic factor for PFS at univariate analysis (HR, 1.422; 95%CI, 1.021–1.980; p-value, 0.037), but it was not confirmed at multivariate analysis. On the other hand, the total dose >30 Gy was a statistically significant negative prognostic factor at univariate analysis (HR, 1.633; 95%CI, 1.174–2.274; p-value, 0.004) and at multivariate analysis (HR, 1.584; 95%CI, 1.032–2.431; p-value, 0.035) (**Table 8**).

**Table 8:** Univariate and multivariate Cox regression analysis of variables predicting PFS on “patient” basis.

		Univariate			Multivariate		
Variable	N.	Hazard ratio	95%CI	p-value	Hazard ratio	95%CI	p-value
**Age, years**							
≤68 >68	89 86	1 0.920	0.663–1.277	0.619			
**Sex** Male Female	112 63	1 1.078	0.767–1.516	0.666			
Burden							
1 lesion More than 1 lesion	112 62	1 1.290	0.917–1.814	**0.143**	1 1.272	0.887–1.824	0.19
Lesion site							
Thorax Pelvis Abdomen Other	81 51 31 12	1 0.565 0.868 0.720	0.381–0.839 0.554–1.360 0.359–1.442	**0.005** 0.535 0.353	1 0.673 0.906 0.729	0.445–1.018 0.573–1.435 0.355–1.496	0.061 0.675 0.388
Total dose							
≤30 Gy >30 Gy	81 94	1 1.633	1.174–2.274	**0.004**	1 1.584	1.032–2.431	**0.035**
BED10							
≤75.9 Gy >75.9 Gy	85 90	1 1.257	0.906–1.745	0.171	1 0.917	0.593–1.419	0.697
CTV							
≤4.82cc >4.82cc	76 99	1 1.422	1.021–1.980	**0.037**	1 1.328	0.941–1.874	0.107
**Dose prescription** ≤97% >97%	104 71	1 0.891	0.639–1.242	0.495			

The rates at 1, 3, and 5 years of OS were 78%, 48%, and 36%, respectively (**Figure 2**). At univariate analysis, having more than one lesion (HR, 1.513; 95%CI, 1.029–2.224; p-value, 0.035), a total dose>30Gy (HR, 1.488; 95%CI, 1.026–2.159; p-value, 0.036), and a CTV>4.82cc (HR, 2.028; 95%CI, 1.378–2.983; p-value, <0.001) were statistically significant negative prognostic factors, but only CTV volume was confirmed at the multivariate analysis (HR, 1.942; 95% CI, 1.294–2.916; p-value, 0.001). On the other hand, a lesion site in the pelvis was a positive statistically significant prognostic factor (HR, 0.455; 95%CI, 0.283–0.730; p-value, 0.001), confirmed at multivariate analysis (HR, 0.544; 95%CI, 0.331–0.894; p-value, 0.016) (**Table 9**).

**Table 9:** Univariate and multivariate Cox regression analysis of variables predicting OS on “patient” basis.

		Univariate			Multivariate		
Variable	N.	Hazard ratio	95%CI	p-value	Hazard ratio	95%CI	p-value
**Age, years** ≤68 >68	89 86	1 0.982	0.681-1.418	0.924			
**Sex** Male Female	112 63	1 1.155	0.791-1686	0.455			
Burden							
1 lesion More than 1 lesion	112 62	1 1.513	1.029-2.224	**0.035**	1 1.415	0.953-2.102	0.085
Lesion site							
Thorax Pelvis Abdomen Other	81 51 31 12	1 0.455 0.871 0.863	0.283-0.730 0.531-1.429 0.412-1.808	**0.001** 0.584 0.697	1 0.544 1.025 0.840	0.331-0.894 0.615-1.708 0.399-1.769	**0.016** 0.925 0.646
Total dose							
≤30 Gy >30 Gy	81 94	1 1.488	1.026-2.159	**0.036**	1 1.208	0.809-1.803	0.356
**BED10** ≤75.9 Gy >75.9 Gy	85 90	1 1.124	0.778-1.623	0.533			
CTV							
≤4.82cc >4.82cc	76 99	1 2.028	1.378-2.983	**<0.001**	1 1.942	1.294-2.916	**0.001**
**Dose prescription** ≤97% >97%	104 71	1 1.071	0.741-1.548	0.715			

*Toxicity.* Treatment was well tolerated. The acute toxicities were nine (4%) cases G1, in the form of pain, fatigue, nausea, vomiting, and cough; one case (0.44%) of anemia G2; and one case (0.44%) of death caused by esophageal bleeding, G5, which happened few days after the treatment on the subcarenal lymph nodes treated with a dose of 45 Gy in three fractions. The only late toxicity was a case G2 (0.44%) in the form of dyspnea.

## Discussion

Today, there is a new stage that is OMD, and emerging data suggest that ablative therapy may improve outcomes and provide durable disease control in this clinical setting (19, 20). This is a study with a large group of patients with OMD treated on NMs by SBRT. The first aim of our study was to evaluate the LC, which was obtained in 90% of cases with rates at 1, 3, and 5 years of 93%, 86%, and 86%, respectively. Comparing these results with the current literature, we can observe an important heterogeneity: 1-year LC rate can range from 95% in the experience reported by Burkon et al. (21), to 82% in the one described by Jereczek-Fossa (22) or 75% in the one reported by Franzese (23). Similar results were reported in terms of 2, 3, or 4 years LC (21–24). These differences in results may depend on various factors, such as the different proportion in terms of primary, radiosensitive vs. radio resistant lesion, and differences in terms of treatments. For example, in the study of Jereczek-Fossa et al., there was a high proportion of colorectal cancers, which are considered less sensitive to radiotherapy; in addiction, the concomitant systemic therapy was not allowed as also in the study of Burkon. In our cohort, the primaries most represented are lung and prostate cancer. In the first case, a better synergic action between SBRT and systemic therapy played a major role; in the second case, we have a series of lesions more radiosensitive in nature (10, 21, 22). Another consideration is that in the study of Franzese et al., the oligoprogressive lesions were at higher risk of local progression following SBRT vs. oligorecurrents. The inferior response in oligoprogressive disease could be potentially correlated to a resistance acquired from previous treatments. In our cohort, the lesions were treated mostly in an oligorecurrence status (23).

In terms of prognostic factors, we registered worse results at univariate analysis in terms of LC with a total dose > 30 Gy. On the contrary, previous studies showed that with higher doses of SBRT, the LC was also higher (25, 26). This result could be explained with our over-mentioned population heterogeneity: as shown in **Table 2**, the lesions in the pelvic or abdominal district were generally treated with a total dose <30 Gy and with prostate as primary tumor. On the other hand, lesions treated in the thoracic district were generally treated with higher doses, and the primary was lung cancer, whose natural history is generally worse than prostatic cancer. These differences could have heavily influenced the univariate and multivariate analysis for LC and other outcomes, explaining some of these unprecedented results.

Another major concern in this clinical setting is the progression of disease in others lymph nodes or in others organ; in the current literature, the authors described similar parameters with slightly different definitions. For example, in our series, we have analyzed LRNC and DNC in the same way of the retrospective study of Franzese et al., and also in this case, we have noticed a heterogeneity of results. In fact, at 1 year, Franzese et al. have recorded a rate of 71% vs. 88% of our study for LRNC and 82% vs. 92%, respectively, for DNC. On the contrary, analyzing the predictive factors of response in our study, the volume of CTV was a statistically significant positive factor of DNC at univariate and at multivariate analysis as the study of Franzese et al. (23).

For the parameter DMFS in our study, the rates at 1 and 3 years of DMF were 57% and 40%, respectively. In the current literature, we have appreciated at 1-year rates that ranged from 67% to 82% (23, 27), and at 3 years, Franzese et al. have showed a rate of 58% (27). In our analysis, the volume and the tumor burden with more than one lesion were a statistically significant negative predictive factor of response in terms of DMFS at univariate and multivariate analyses. Similar results were found in other retrospective and prospective studies, where the increased disease load, as volume and/or as number of lesions, can affect increased metastatic cascade and the risk is significant (28, 29).

The rates of PFS in our study at 1 and 3 years were 44% and 23%, respectively; in the literature, described rates ranged between 28% and 95% at 1 year and from 17% and 22% at 3 years (13). In our analysis, pelvic lesions were correlated with better results in terms of PFS and OS; the same was recorded by Franzese et al. who treated abdominal-pelvic NMs with SBRT with a rate of PFS at 5 years of 73%. In our analysis, we had better LRNC and DNC when the lesion site was in the abdomen (8). As described above in **Table 2**, the differences in terms of histology could have played an important role in our analysis.

In our study, the rates at 1, 3, and 5 years of OS were 78%, 48%, and 36%, respectively; data were almost confirmed in some studies with rates at 1, 3, and 5 years of 76%, 49%, and 41%, respectively (7, 22, 30). In our analysis, the volume and the tumor burden with more than one lesion were a statistically significant negative predictive factor of response in terms of OS at the univariate analysis, and similar results were found in others studies (29, 30).

In general, the most challenging site for NMs SBRT seemed to be the thorax with worst LC, PFS, and a major risk of fatal complication (3–34). In fact, also in our study, we had a case of death for an NM treated in the mediastinum. In the systematic review of Deodato et al., they concluded that considering all the patients included in the studies, the rates of G ≥ 3 were 2% and G5 toxicities were 0.2%, respectively (13). In our study, except for a toxicity G5, we had no G3 toxicity; therefore, we can conclude that SBRT is a treatment well tolerated.

This experience represents one of the largest populations in this clinical setting to the best of our knowledge, even though its retrospective nature and its heterogeneous population in terms of primary and lesion sites represent an important limit. It is probably due to this high level of heterogeneity, especially in terms of doses and fractionations, that no relation between BED and outcomes was found. This underlines the necessity of the patient selection for this type of treatment and the definition of the ablative doses, which are crucial. Technology is also an important bias that must be considered in this type of experiences; however, in this series, the patients were treated with the same type of technology from 2007 up to the end of 2022.

A metastasis direct therapy as SBRT on NMS is a very important therapeutic strategy especially with the advent of new systemic therapies whose synergy could give great results. Maybe in the future, with the introduction of circulating DNA, we could have more information about residual tumor, which will help us in choosing an ideal tailored treatment (35).

In conclusion, SBRT is an option for the treatment of NMS, with high rates of LC, improving the data of survival, with a good safety and tolerance. However, prospective studies are necessary to better understand the right cohort of patients to be treated and eventually the integration with systemic therapy.

## Data availability statement

Requests to access the datasets should be directed to the corresponding author.

## Ethics statement

The Internal Review Board of S. Andrea Hospital—La Sapienza University of Rome approved our study. Considering the retrospective design, analyzing existing data, ethical review and approval code from the Ethics Committee was not required in accordance with the local legislation and institutional requirements. Informed consent was obtained from all subjects involved in the study. The patients/participants provided their written informed consent to participate in this study.

## Author contributions

Conceptualization of the study: DC and VDS. Data curation: DC, RS, and MR. Formal analysis: DP, DC, and PB. Performed methodology: DC, DP, and PB. Supervised the study: DC, PB, MO and VDS. Wrote the article: DC. Reviewed the manuscript: DP, PB, and DC. Critical revision and Final approval of the version to be published: DC and VDS. PB and DP authors share second authorship. All authors contributed to the article and approved the submitted version.
